# The Lack of Dopamine Transporter Is Associated With Conditional Associative Learning Impairments and Striatal Proteomic Changes

**DOI:** 10.3389/fpsyt.2022.799433

**Published:** 2022-03-18

**Authors:** Artem Savchenko, Carina Müller, Jana Lubec, Damiana Leo, Volker Korz, Leila Afjehi-Sadat, Jovana Malikovic, Fernando J. Sialana, Gert Lubec, Ilya Sukhanov

**Affiliations:** ^1^Institute of Pharmacology, Pavlov First Saint Petersburg State Medical University, St. Petersburg, Russia; ^2^Department of Pharmaceutical Chemistry, University of Vienna, Vienna, Austria; ^3^Programme for Proteomics, Paracelsus Medical University, Salzburg, Austria; ^4^Department of Neurosciences, University of Mons, Mons, Belgium

**Keywords:** DAT, proteomic analysis, striatum, associative learning, knock-out animal model

## Abstract

Dopamine (DA) is critically involved in different functions of the central nervous system (CNS) including control of voluntary movement, affect, reward, sleep, and cognition. One of the key components of DA neurotransmission is DA reuptake by the DA transporter (DAT), ensuring rapid clearance of DA from the synaptic cleft. Thus, lack of DAT leads to persistent high extracellular DA levels. While there is strong evidence for a role of striatal dopaminergic activity in learning and memory processes, little is known about the contribution of DAT deficiency to conditional learning impairments and underlying molecular processes. DAT-knockout (DAT-KO) rats were tested in a set of behavioral experiments evaluating conditional associative learning, which requires unaltered striatal function. In parallel, a large-scale proteomic analysis of the striatum was performed to identify molecular factors probably underlying behavioral patterns. DAT-KO rats were incapable to acquire a new operant skill in Pavlovian/instrumental autoshaping, although the conditional stimulus–unconditional stimulus (CS-US) association seems to be unaffected. These findings suggest that DAT directly or indirectly contributes to the reduction of transference of incentive salience from the reward to the CS. We propose that specific impairment of conditional learning might be caused by molecular adaptations to the hyperdopaminergic state, presumably by dopamine receptor 1 (DRD1) hypofunction, as proposed by proteomic analysis. Whether DRD1 downregulation can cause cognitive deficits in the hyperdopaminergic state is the subject of discussion, and further studies are needed to answer this question. This study may be useful for the interpretation of previous and the design of future studies in the dopamine field.

## Introduction

Although dopamine (DA) neurons are comparatively few in number (a total of around 400,000–600,000 in the human brain (1–3), DA is critically involved in different functions of the central nervous system related to the control of voluntary movement, affect, reward, sleep, and cognition (4, 5). Relatively small populations of the DA neurons, arising from the substantia nigra pars compacta and the ventral tegmental area, modulate the activity of the striatum, which is functionally coupled with the frontal cortex and essential for different kinds of learning (6–13). The dopaminergic modulation of frontostriatal circuit provides the ability to adapt learning processes on an organism's needs, motivation, and reward history (14).

The DA transporter (DAT), a member of the Na^+^/Cl^−^-dependent transporter family selectively expressed in dopaminergic neurons, critically regulates DA neurotransmission by transporting extracellular DA into the intracellular space (15). Changes in DAT expression have been reported in patients with a number of neuropsychiatric diseases characterized by cognitive deficits including schizophrenia, ADHD, and Parkinson's disease (16–18). However, whether these findings are essential for the pathogenesis of cognitive disturbances is still unknown.

A strain of DAT-KO rats with a loss-of-function mutation in the *Slc6a3* gene was developed recently using a zinc finger nuclease technology (19). Animals lacking DAT are characterized by a high striatal concentration and persistent extracellular DA but markedly reduced total tissue DA levels (19, 20). Peculiar phenotypic features are linked to the changes in DA levels. Comparable to DAT-KO mice (21), DAT-KO rats weigh less than heterozygote (HT) and wild-type (WT) rats, demonstrating dramatically increased locomotor activity and prominent motor and oral stereotypies (19, 22). Mutant rats also display impaired working memory (8), changes in learning an object recognition task (23), and altered male sexual behavior (24).

Thus, the DAT-KO rats are a promising model for studying frontostriatal DA pathway dysfunctions. However, the conditional associative learning processes in the mutant rats have not been studied yet as well as the molecular mechanisms. Therefore, we aimed to evaluate the effect of DAT disruption in the conditional associative learning process together with striatal proteomic analysis. We report that DAT deficiency leads to impairment of associative learning process that requires an assignment of incentive salience to conditioned stimuli (CS). These alterations occur with disturbances of synaptic transmission, axo-dendritic transport and DA-binding processes presumably related to DA receptor 1 (DRD1) downregulation.

## Methods

### Animals

All experiments were performed in the animals originated from the previously described rat strain with a loss-of-function mutation of the DAT gene (19). Drug and experimentally naïve male DAT-KO rats as well as their HT and WT littermates (2–3 months old and weighing 200–400 g at the beginning of the experiment) from the local colony of Pavlov Medical University (PMU) were individually housed in TIIIH cages (Tecniplast, Buguggiate, Italy) with wood-based animal bedding (Lignocel, BK 8-15, JRS, J. Rettenmaier & Söhne Group, Rosenberg, Germany) under a 12-h/12-h light/dark cycle (lights on at 08:00 h) at 21 ± 2°C and 50 ± 20% humidity. The animals had free access to filtered (“AQUAPHOR,” Saint Petersburg, Russia) tap water. The cages, bedding, and water bottles were changed once a week. We restricted the animal food consumption before the start of the experiments so that their body weight decreased approximately to 85% of the initial one. During the experiments, the amount of food per day (15–16 g for WT and HT and 20 g for DAT-KO) was set so that the body weight gain was limited by 2–3 g per week.

### Behavioral Experiments

**Figure 1:**
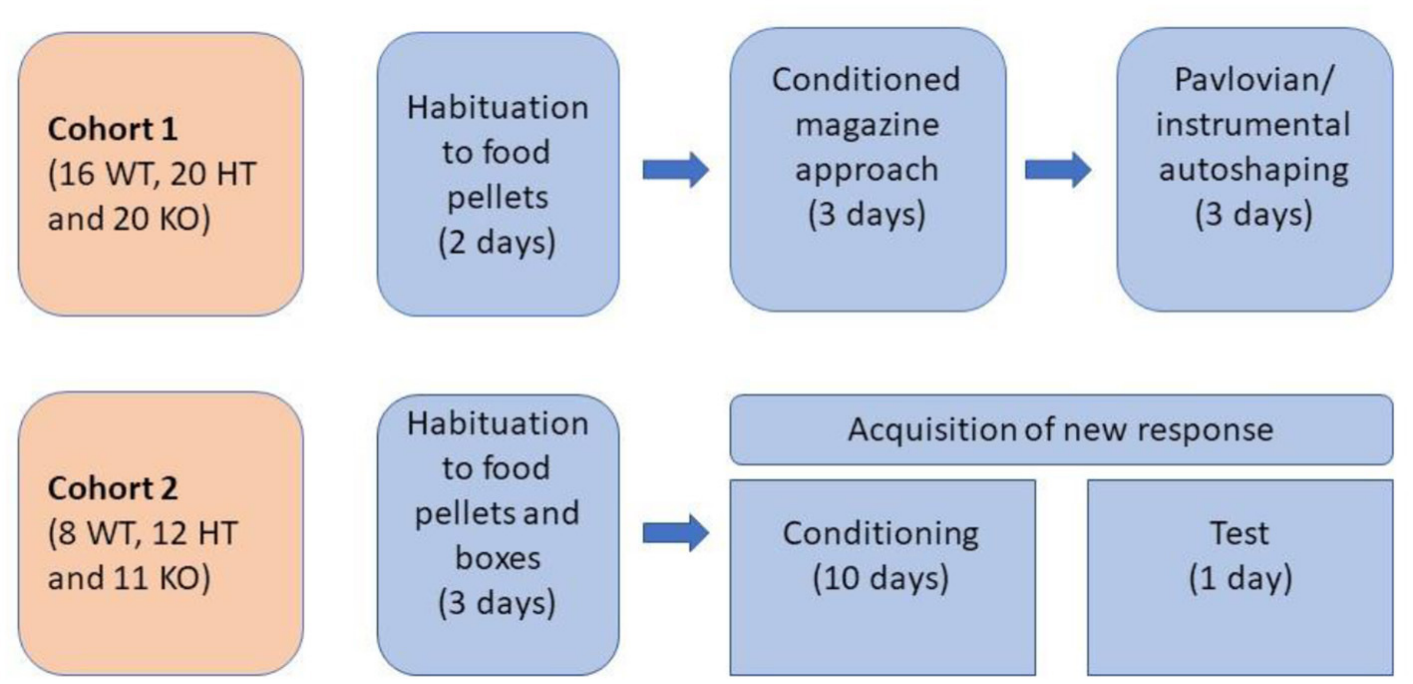
Design of behavioral testing.

All behavioral experiments were carried out during the light period of the light/dark cycle after at least 1 week of habituation to the animal facility. Experimental protocols were approved by the local Animal Care and Use Committee of PMU. The distribution of rats in two experimental groups and the order of the tasks are presented in Figure 1.

#### Apparatus

The experiments were performed in six standard modular operant conditioning chambers for rats (interior dimensions: 30.5 ×24.1 ×29.2 cm; ENV-007, MED Associates Inc., East Fairfeld, VT, USA) placed in ventilated, light-proof, and sound-attenuated boxes with an electric fan providing air circulation and a background white noise. A pellet tray (ENV-200R2MA) was set up at 2 cm from the floor on the middle panel of the right (five chambers) or the left (one chamber) wall. The tray was equipped with a pair of nose-poke photobeam infrared sensors (ENV254-CB). A pellet dispenser (ENV-203-45) was located outside of the chambers, and 45-mg food pellets (P.J. Noyes Inc., Lancaster, New Hampshire, USA) were delivered into the tray. A speaker (ENV-223AM) was situated under the tray, and a white house light (ENV-215M) was placed at the top of the middle panel. Response devices, either retractable levers (model ENV-112BM; PiAT) or nose-poke holes (model ENV-114AM; ANR), were placed on the sides of the food tray.

#### Conditioned Magazine Approach

48 and 24 H before the testing in the operant chamber, each rat received 15 food pellets in the home cage to habituate them to pellet consumption. We started the operant box test only when the animals have eaten all the given pellets.

**Figure 2:**
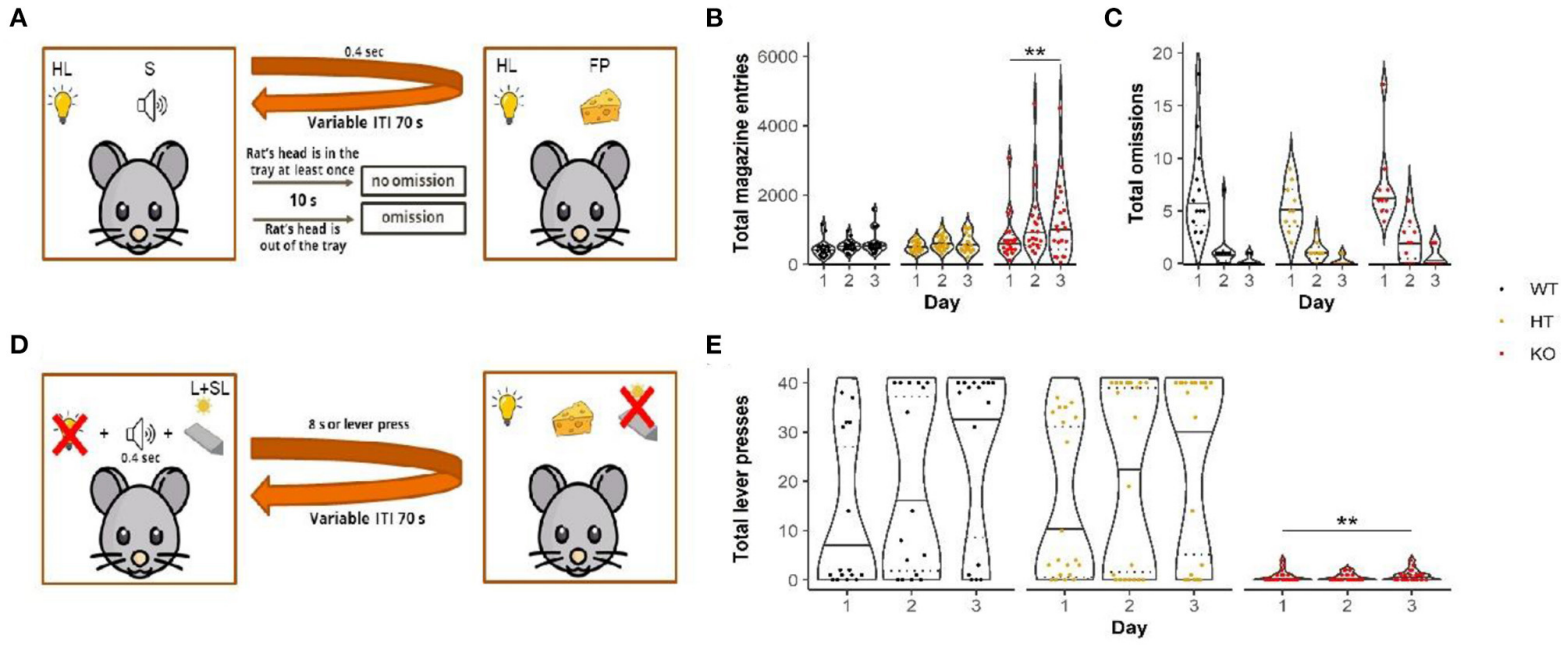
Conditional magazine approach task **(A)** revealed elevation in appetitive behavior indicated as more magazine entries made by mutants **(B)** without affecting conditioning (number of omissions) **(C)**. In the Pavlovian/instrumental autoshaping procedure, **(D)** DAT-KO rats displayed pronounced impairments demonstrated by significantly reduced number of lever presses **(E)**. Data are represented as violin plots with median (solid line) and 25^th^ and 75^th^ percentiles (dotted lines) and points indicating each value. HL, house light; S, sound; FP, food pellet; ITI, intertrial interval; L+SL, lever + signal light; **p <0.01, Dunn's test.

The experimental procedure was adapted from Choi et al. (25) (Figure 2A). The start of an experimental session was indicated by the house-light illumination. During each of the three daily sessions, 28 pellets were delivered individually into the food tray on the variable-time 70-s (30-110-s) schedule. Each food pellet administration was paired with a sound stimulus (400 ms, 78 dB). If a rat's head was out of the tray during 10 s following the pellet administration, the trial was scored as “omitted.” The total number of omissions and the total number of tray entries were controlled and recorded automatically by computer.

#### Pavlovian/Instrumental Autoshaping

We used the experimental procedure adapted from the previous work (26) (Figure 2D). The procedure included three daily sessions. The house-light illumination indicated start of each experimental session. The session consisted of 40 trials divided by a variable inter-trial period, the duration of which was on average 70 s (30-110 s). At the beginning of each trial, a rat got a complex stimulus (the house light turning off, the lever introduction into the operant chamber, and signal yellow light above the lever). Assignment of levers (left/right) was counterbalanced across subjects. The stimulus continued for 8 s followed by food pellet delivery with a sound signal (400 ms, 78 dB). However, if a rat pressed the lever, the complex stimulus was ended (the house light turning on, the lever retraction, and signal yellow light turning off) and the pellet was delivered immediately. The number of the performed lever presses was automatically recorded.

#### Acquisition of New Response Reinforced by CS

**Figure 3:**
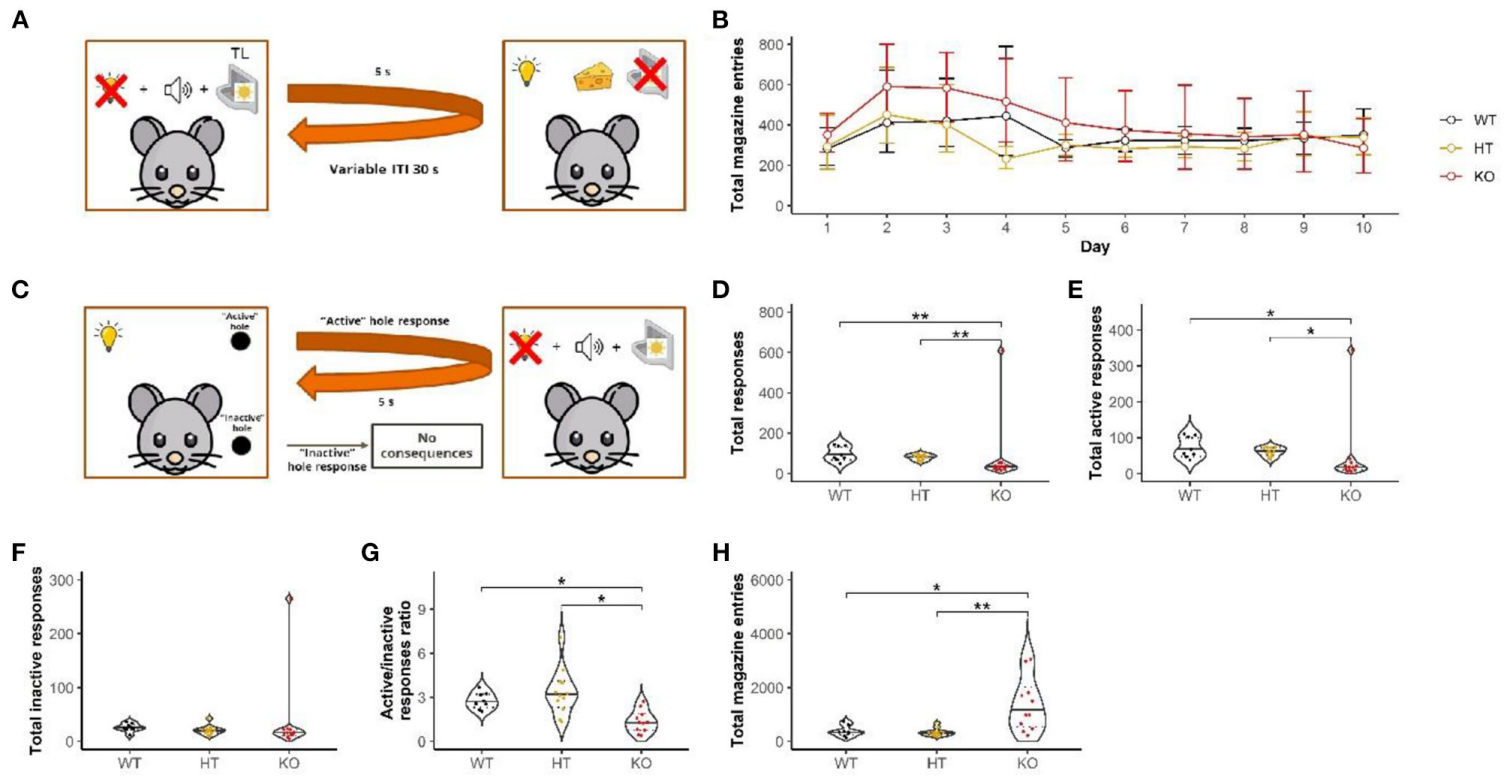
Acquisition of new response reinforced by CS was disrupted in the DAT-KO rats. Although throughout the first stage **(A)** conditioning was unaffected **(B)**, during the test **(C)** active hole preference was lower for DAT-KO rats **(G)** without difference in responding in the “inactive” hole **(F)**. Data are represented as mean ± 95% confidence interval **(B)** and violin plots with median (solid line) and 25th and 75th percentiles (dotted lines) and points indicating each value **(D–H)**. TL, tray light; *p <0.05, ***p <0.001, Dunn's test.

The experimental procedure was adapted from the previous study (27) (Figures 3A,C). The rats underwent a 1-h session for three consecutive days in order to familiarize with the operant chamber and food pellets. The conditioning phase consisted of ten sessions (one session per day). The subjects received 30 pairings of a complex 5-s stimulus (house light off, tray light, and 78-dB sound on) followed by delivery of a food pellet within the course of each session. The pairings were delivered on the random time 30-s schedule. The animals did not have access to the nose-poke openings during the habituation and conditioning phases. During this phase, the PC recorded the total number of magazine entries into the tray.

Following conditioning, we tested the animals in the secondary reinforcement paradigm. In this phase, the rats finally got access to nose-poke holes. The response into one hole (“active”) resulted in the presentation of the 5-s complex stimulus only (without food reward). Responding into the other one (“inactive”) had no programmed consequences. The position (left/right) of the “active” hole was counterbalanced across subjects. The session lasted for 60 min.

We used the following parameters for the analysis: (1) the total number of nose pokes into the holes, (2) the total number of “active” nose-poke responses; (3) the total number of the “inactive” nose-poke responses; (4) the response ratio as the total number of the “active” nose-poke responses divided by the total number of the “inactive” nose-poke responses; (5) the total number of nose-poke responses into the magazine tray; and (6) the total latency of tray check following stimulus administration.

#### Statistical Analysis

Non-parametric statistical methods [the Kruskal–Wallis test and the mixed-design analysis of variance on ranked data (ANOVA)] were used to analyze behavioral data. Dunn's and Bonferroni's *post-hoc* tests were performed whenever significant results were indicated by the abovementioned statistical methods. Alpha was set at 0.05. SigmaPlot 12.5 (Systat Software Inc., San Jose, CA, USA) or IBM SPSS Statistics 21 (IBM, Armonk, New York, USA) were used for analysis.

### Proteomics Quantification of Striatal Fraction From DAT-KO and WT Rats

Different groups of naïve rats (DAT-KO and WT) were used for proteomic analysis. The rats were exposed to carbon dioxide and decapitated by guillotine. The brains were rapidly removed, and the striatum was dissected on a Para Cooler (RWW Medizintechnik, Hallerndorf, Germany) at 4°C. The tissue was immediately stored at −80°C until proteomic analysis. Protein sample preparation and LC-MS/MS was performed as previously described (28). All homogenization and centrifugation steps were carried out on ice and at 4°C. Brain tissues were homogenized in an ice-cold homogenization buffer [10 mM HEPES, pH 7.5, 300 mM sucrose, 1 × Protease Inhibitor Cocktail (PIC, Roche Molecular Biochemicals, Pleasanton, CA, USA)] using a Dounce homogenizer; the homogenate was centrifuged at 1,000 × g for 10 min to remove cell debris and nuclei, and the supernatant was collected. The pellet was resuspended again in the homogenization buffer and centrifuged at 1,000 × g for 10 min. The pooled supernatants were then centrifuged at 15,000 × g for 30 min to obtain the total membrane fraction enriched in synaptosomes and mitochondria. The resulting pellets were washed with 10 mM HEPES, pH 7.5, PIC, and solubilized in 50 mM TEAB buffer (Sigma-Aldrich), 7 M urea, 2 M thiourea, 4% CHAPS, 100 mM DTT, and PIC. The protein concentration was determined by Pierce™ 660-nm Protein Assay (Thermo Scientific, Waltham, MA, USA).

Protein samples were digested 18 h with trypsin (Promega Corporation, Madison, WI, USA) using filter-aided sample preparation (FASP) (29) with 70 μg of protein per one reaction. Tryptic peptides were desalted using reversed-phase C18 stage tips (30) and reconstituted in 40 μl of 100 mM TEAB (Sigma-Aldrich, St. Louis, MO, USA). The actual amount of peptides was determined by Pierce™ Quantitative Fluorometric Peptide Assay (Thermo Scientific). A volume corresponding to 6 μg of peptides was transferred from each sample into separate vial, dried at 30°C (SpeedVac, Eppendorf, Framingham, MA, USA), and reconstituted in 17 μl of 0.1% formic acid. The peptides (5 μl injection volume) were separated by LC using the following gradient of solvent A (2% acetonitrile, 0.1% TFA in water) and solvent B (80% acetonitrile in 0.1% water) [0–7.2 min 5% B; 7.2–230 min 5%-30% B; 230–250 min 30%-50% B; 250–255 min 90% B; 255–260 min 5% B]. MS analysis was performed by the Thermo Scientific™ Q Exactive™ Plus Orbitrap mass spectrometer (Thermo Scientific) in positive ion mode with the following settings: full-scan MS in the range of m/z 350–1500 at the resolution of 140,000 (at m/z 200). MS/MS scans were acquired at the resolution of 17,500 (m/z 200) through HCD fragmentation of 20 most intense ions at 27% normalized collision energy with a fixed mass of 100 m/z.

Raw data were analyzed by MaxQuant 1.6.17.0 using the Andromeda searching engine and LFQ algorithm (31). Data were searched against *Rattus norvegicus* UniProt sequence database (downloaded on March 29th, 2021; 29936 entries) using the following search parameters: carbamidomethylation of cysteine as fixed modification; oxidation of methionine and protein N-terminal acetylation as variable modifications; trypsin as proteolytic enzyme with maximal two missed cleavages; a second peptide option used; 20 and 4.5 ppm of peptide mass error tolerances for first search and second searches, respectively; and a minimum of 7 aa per a peptides. For identification, parameters were set to 0.01 PSM FDR, 0.01 protein FDR, and 0.01 site decoy fraction.

Averaged values from technical replicates were used for quantification. All data were analyzed by Perseus software (32) as follows. The data set was filtered for proteins with a minimum of 4 valid values (≥2 unique peptides) in at least one group (DAT-KO or WT). LFQ intensities were log2-transformed, and missing values were imputed using a downshifted normal distribution (width 0.3, downshift 1.8). Protein groups with p <0.05 (unpaired *t* test) and fold change >1.5 were considered significantly changed.

For the heat map, the log2-transformed LFQ intensities were z-scored and the hierarchical clustering was computed using the Euclidean distance (33). Gene ontology (GO) analysis was performed using the ClueGO Cytoscape plug-in (34). ClueGO parameters were set as indicated: Go Term Fusion selected; only display pathways with *p-*values ≤ 0.05; GO tree interval 3–10 levels; GO term minimum # genes, 3; threshold of 4% of genes per pathway. The statistical test used for the enrichment was based on the right-sided hypergeometric test with a Benjamini–Hochberg correction and kappa score of 0.4.

The mass spectrometry proteomics data have been deposited to the ProteomeXchange Consortium *via* the PRIDE (35) partner repository with the dataset identifier PXD028157.

## Results

### Behavioral Experiments

#### Pavlovian Conditioning Seems to Be Unaffected in DAT-KO Rats

All rats were successfully habituated to food pellet consumption and ate all pellets while being tested in the operant boxes.

Due to the program inaccuracies in counting omissions, part of data was excluded from analysis of this parameter, whereas the number of magazine entries was not affected. The number of omissions decreased from the 1st to the 3rd day, meaning that animals from all three groups learned the task (Figure 2C). Statistical analysis (mixed-design ANOVA on ranks) revealed the significant main effect of factor “day” [*F*_(2, 50)_ = 155.9, *p* <0.001; Bonferroni's test: 1^st^ vs. 2^nd^ day, 1^st^ vs. 3^rd^ day, 2^nd^ vs. 3^rd^ day *p* <0.001]. However, no differences were found among the different genotypes [*F*_(2, 37)_ = 1.9, *p* = 0.16]. The analysis also failed to reveal the significant effects of factors “day” and “genotype” interaction [*F*_(4, 50)_ = 0.14, *p* = 0.97].

Throughout training days, the number of magazine entries increased [the effect of factor “day”: *F*_(2, 64)_ = 12.7, *p* <0.001] with the greater number of those observed on the 2nd and 3rd days (Bonferroni's test: 1st vs. 2nd day, 1st vs. 3rd day *p* <0.01) (Figure 2B). Moreover, the number of entries was affected by genotype [the effect of the factor “genotype”: *F*_(2, 54)_ = 3.9, *p* <0.05]. The DAT-KO rats performed significantly more entries than the WT rats (Bonferroni's test: *p* <0.05). However, no significant interaction between “day” and “genotype” factors could be detected [*F*_(4, 64)_ = 0.6, *p* = 0.67].

#### Impaired Pavlovian/Instrumental Autoshaping in DAT-KO Rats

We observed an apparent difference between the different genotypes in the number of lever presses (Figure 2E). While the number of the WT and HT rats' responses increased from the 1st to 3rd days of training, the number of the DAT-KO rats lever pressing was consistently lower; none of the mutant animals performed more than five responses during any session. Statistical analysis (mixed design ANOVA on ranks) revealed a significant main effect of days [*p*_(2, 51)_ = 9.5, *p* <0.001; Bonferroni's test: 1st vs. 3rd day *p* <0.001] and the significant main effect of genotype [*F*_(2, 46)_ = 11.8, *p* <0.001; Bonferroni's test: DAT-KO vs. WT and HT *p* <0.01]. We did not find any significant interaction between days and genotype [*F*_(4, 51)_ = 1.9, *p* = 0.13].

#### Acquisition of New Response Reinforced by CS Is Disrupted in Rats Lacking the DAT

All animals successfully proceed to the Pavlovian conditioning. During the conditioning stage, DAT-KO rats did not show differences in the total number of magazine entries (mixed design ANOVA on ranks: “genotype” *F*_(2, 27)_ = 1.2, *p* = 0.3; “day” *F*_(9, 39)_ = 1.6, *p* = 0.17; “genotype” by “day” interaction *F*_(18, 39)_ = 1.2, *p* = 0.3; Figure 3B) compared to HET and WT.

However, we found a genotype effect in the secondary reinforcement paradigm. In the test session, DAT-KO rats exhibited a lower number of responses in both holes (H-test: H = 13.3, df = 2, *p* <0.01; Dunn's test: DAT-KO vs. WT and DAT-KO vs. HT *p* <0.01; Figure 3D). Notably, DAT KO rats displayed significantly less responses in the “active” hole than both the HT and WT littermates [the Kruskal–Wallis test (H-test): H = 13.2, df = 2, *p* = 0.001; Dunn's test: DAT-KO vs. WT and DAT-KO vs. HT *p* <0.05; Figure 3E]. The lack of DAT did not affect responses into the “inactive” hole (H-test: H = 5.6, df = 2, *p* = 0.06; Figure 3F). The WT and HT rats, but not the DAT-KO littermates, preferentially visited the active hole as shown by the ratio of “active” responses/”inactive” responses (H-test: H = 15.6, df = 2, *p* <0.001; Dunn's test: DAT-KO vs. WT and DAT-KO vs. HT *p* <0.05; Figure 3G). For the DAT-KO rats, the number of magazine entries was significantly higher (H-test: H = 12.3, df = 2, *p* <0.01; Dunn's test: DAT-KO vs. WT *p* <0.05 and DAT-KO vs. HT *p* <0.01) (Figure 3H).

### Proteomic Results

**Figure 4:**
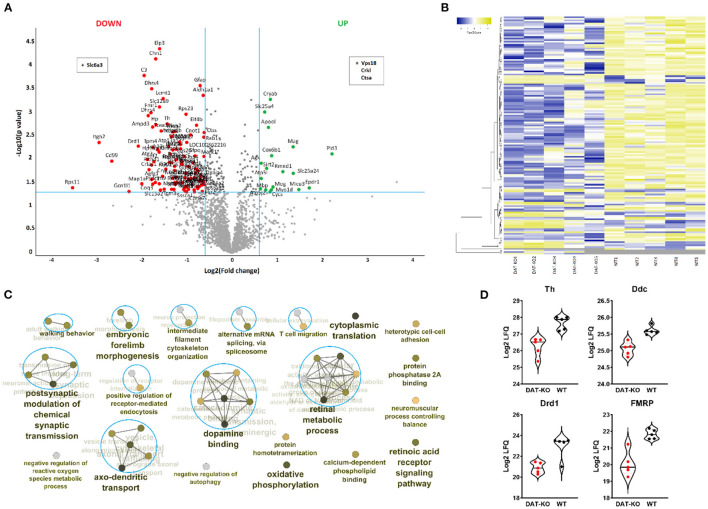
Enriched synaptosomal striatal fractions from DAT-KO rats and WT were subjected to label-free quantitative proteomic analysis. **(A)** A volcano plot showing proteins upregulated (green) and downregulated (red) in the DAT-KO group compared to WT (*p* <0.05, fold-change > 1.5). The protein groups in the upper middle section (black) are those which fulfilled the requirement for the *p*-value cut-off (*p* <0.05) but did not match the fold-change cutoff. **(B)** A hierarchical clustering heatmap is presented for the differentially expressed proteins (*p* <0.05, fold change > 1.5; *n* = 5). **(C)** The clustered network map of enriched gene ontology (GO) Biological Processes and Molecular Function terms on the list of significantly altered proteins between the groups. Enriched GO terms consisting of various related genes are depicted as nodes. The FDR q-value of each GO-term is color-coded. Displayed GO terms are the most significant cases from significantly enriched clusters. The connectivity (edges) between the terms in a functionally grouped network is derived from kappa score, which indicates the similarity of associated genes shared by different terms. **(D)** The violin plots show protein expression levels of representative differently expresses proteins.

Label-free quantification was used to identify proteomic differences in the striatum between the DAT-KO and WT rats (*n* = 5). A total number of 2,210 protein entries were unambiguously identified and used for quantification (>2 unique peptides in at least 4 biological replicates in at least 1 group). A total number of 126 protein groups were significantly different between treatment groups (*p* <0.05, fold-change > 1.5). Of these, 108 proteins were downregulated and 18 proteins upregulated in the DAT-KO compared to the WT animals (Figure 4A). Four proteins, including DAT (Slc6a3), were identified only in one group. A hierarchical clustering heatmap shows differently expressed proteins in all samples (Figure 4B). All significantly different proteins between treatment groups are listed in the Supplementary Table 1.

To translate proteomic differences in the DAT-KO into functional consequences, functional enrichment analysis with significantly different proteins was performed using the Cytoscape plug-in ClueGo (Figure 4C). Forty four significantly enriched GO terms were categorized *via* their shared genes into 20 GO groups. As expected, DA-related processes were altered in the DAT-KO rats. Moreover, altered proteins were enriched in axo-dendritic transport, postsynaptic modulation of chemical synaptic transmission, regulation of receptor-mediated endocytosis, etc., whereas the majority of associated proteins were downregulated in the rats with the lack of DAT. All significantly enriched GO terms and identified associated proteins are listed in the Supplementary Table 1.

## Discussion

In the present study, we applied different approaches to evaluate conditional associative learning processes in DAT-KO rats. We observed that the DAT-KO rats are characterized by impaired capability to learn new stimulus–response associations. The behavioral alteration here reported might be caused by specific molecular adaptations (first of all, DRD1 downregulation) as revealed in the DAT-KO rats' striatum by proteomic analysis.

### Hyperdopaminergia Is Associated With Specific Impairments of Conditional Learning

We used the conditioned magazine approach task to assess the ability of the rats to associate conditioned (CS) and unconditioned (US) stimuli. We did not detect any differences in the number of omissions between the KO and control (WT and HT) animals at any experimental day. The number of omissions decreased in all three groups from the 1st to the 3rd session/day; thus, Pavlovian conditioning seems to be unaffected by the depletion of DAT in rats. The elevation in goal-directed performance (the increased number of the magazine entries) might support the ideas of an altered inhibitory control and of an increased “wanting” of the DAT-KO rats, described previously in hyperdopaminergic mice (36, 37). We can speculate that the unchanged number of omissions is possibly due to these phenomena and do not necessarily demonstrate the absence of cognitive impairments.

In line with previous studies (37), we observed an increased number of magazine entries in DAT-KO rats. This increase can be due to DAT-KO hyperactivity (19). However, it may also be linked to enhanced acquisition of conditioned responding associated with increased DA brain levels. For example, Phillips et al. reported that DAT inhibition by administration of *d*-amphetamine in the nucleus accumbens resulted into the facilitation of appetitive Pavlovian conditioning (38).

The Pavlovian/Instrumental autoshaping task is an informative tool for evaluation of learning and memory processes in rodents (39). In the present study, the DAT-KO rats were completely unable to acquire a new operant skill in this task. Taking into account the unaltered (or even facilitated) DAT-KO rat US-CS association, we hypothesize that DAT depletion and related decreased DRD1 levels can set the goal-tracking (magazine approach) strategy represented by interactions with the reward tray during the CS. It has been proposed that stimulus–reward associations that produce different CRs are mediated by different neural circuitries (40). An intact DA transmission is generally required only for sign-tracking strategy in which the reward cue acquires powerful motivational properties (40). Thus, alteration of DA signaling in the DAT-KO rats seems to reduce the transfer of incentive salience from the reward to the CS. Nevertheless, we cannot exclude other explanations, like meaning of the previous Conditioned magazine approach task experience.

Flagel et al. (41) showed that novelty-seeking behaviors can be predictive for a behavioral strategy involved in Pavlovian conditioning: high responders to novelty consistently learn a sign-tracking CR whereas low responders to novelty consistently learn a goal-tracking CR. However, there are controversial studies for the DAT-KO rats in novelty detection (23, 42).

The findings in the Pavlovian/Instrumental autoshaping task might be explained by both impaired incentive salience assignment and disturbances of instrumental behavior acquisition in the KO animals. To discriminate about them, we evaluated the ability of CS to get reinforcing properties by itself (i.e., to become a secondary/conditioned reinforcement and get incentive value) in rats. This process seems to be dramatically disrupted in the DAT-KO rats. It is worth noting that CS can acquire incentive value only in “sign-trackers” (43), so these results are in accordance with output of the previous test. Moreover, DAT-KO rats did not differ from their WT and HT littermates in the number of magazine entries during conditioning stage. These findings support the result of the conditioned magazine approach task and allow us to exclude unsuccessful Pavlovian conditioning in the rats. Interestingly, mice with decreased levels of DAT did not demonstrate impairments in the Pavlovian-to-instrumental transfer task (37). This discrepancy can be explained by differences in the degree of DAT expression (0% in the KO rats vs. 10% in the KD mice) associated with a corresponding different increase in synaptic DA concentration (2- vs. 5–7-fold), as well as by different experimental methods or differences between the rodents tested.

Taken together, our behavioral findings demonstrate that the associative learning seems to be very sensitive to DA neurotransmission induced by the DAT depletion.

Our data could also have a translational value: patients with schizophrenia, characterized by prefrontal DA hypofunction and striatal DA hyperfunction (44), are not able to differentiate between salient and non-salient stimuli (45). In the same study, patients demonstrated to still have CS–US conditioning (45). Moreover, the administration of dopaminomimetics seems to be able to disorganize salience in similar ways. Therefore, the DA agonists (pramipexole or ropinirole)-treated patients with Parkinson's disease demonstrated that the aberrant salience assignment resulted in an irrelevant CS–US association (46). Intriguingly, the authors of this study failed to find some distinction in salience attribution between the non-treated patients with nigrostriatal DA hypofunction and controls.

It is worthy to note that the Pavlovian/instrumental autoshaping task and the acquisition of a new response reinforced by the CS task appear to be less sensitive to motor dysfunctions than the conditioned magazine approach task because of the prolonged habituation to experimental settings. Nevertheless, we cannot exclude the significance of this factor that can limit the theoretical value of the results.

### DAT Deficiency Is Accompanied by Striatal Protein Changes

The impaired behavioral performance was associated with modifications in striatal protein expression closely related to learning and memory mechanisms (i.e., synaptic transmission, axo-dendritic transport, and DA-binding processes).

The DAT-KO animals show major changes in the DA system, including altered DA synthesis, high extracellular DA levels, and impaired DA receptor density (47). Dopaminergic neurotransmission begins at the biosynthesis step, with tyrosine hydroxylase (TH) the rate-limiting enzyme for DA biosynthesis. As previously demonstrated (48, 49), here we show that DAT loss is associated with decreased TH protein levels (Figure 4D). Moreover, in DAT KO rats, we also found decreased protein levels of DOPA decarboxylase, the enzyme responsible for the synthesis of DA from L-DOPA (Figure 4D). It is well known that increased extracellular DA amount can inhibit DA synthesis through its autoreceptor activation by at least 50% (50).

Next, DRD1 protein expression was significantly decreased in DAT-KO compared to WT (Figure 4D). These results confirm previous studies on DAT-KO rats and mice (19, 51). Receptor internalization is often observed in condition of elevated levels of extracellular DA or DA receptor agonism (51, 52) and other brain regions (53). The DRD1-reduced expression may be also responsible for the developmental changes in the DAT-KO rats. Illiano et al. (54) found alterations in prefrontal cortex neurotransmission, signs of neurodegeneration, and glial activation during early adolescence in the rats with the lack of DAT.

DRD1 hypofunction might explain the impaired performance of the DAT-KO rats in the Pavlovian/Instrumental autoshaping task since the sign-tracking strategy was shown to be under the control of DRD1-expressing neurons (55). DRD1 inhibition specifically prevents the acquisition of the sign-tracking to a lever, instead promoting the goal-tracking strategy (56), probably through degradation of the motivational properties of the CS, which are required for the CS to become attractive. However, DRD2 may also play a role in the sign-tracking behavior acquisition as indicated by disrupted conditioned responding in both goal- and sign-trackers following D2/D3 agonist and antagonist treatments (43). DRD2/3 agonist 7-OH-DPAT attenuated the reinforcing properties of a lever in sign-trackers (43), which also suggest the DRD2/3-dependent disruption of incentive value acquisition in the hyperdopaminergic state. Decreased levels of DRD2 have been detected in DAT-KO rats (19); however, we were not able to unambiguously quantify DRD2 levels.

In terms of dopaminergic signaling, fragile X mental retardation protein (FMRP) has been shown to be critically involved in modulation of DRD1-mediated inhibition in the prefrontal cortex (57) and serves as a mediator of cocaine-induced behavioral and synaptic plasticity in NAc (58). In the current study, the protein levels of FMRP were decreased in the mutant animals (Figure 4D).

Moreover, some studies highlighted the possibility that DA is involved in oligodendrocyte development and myelin formation, in particular through DRD2 or DRD3. Chronic treatment with the DRD2 antagonist haloperidol reduces the expression of myelin protein in mice (59), whereas activation of DRD2 increases the number of oligodendrocyte progenitor cells. The increased levels of major myelin proteins (Mbp, Mag, and Mog) in DAT-KO may indicate augmented myelination as a result of hyperdopaminergic conditions.

Impairment of autophagy, a major eukaryotic cell clearing machinery, may occur following abnormal stimulation of DA receptors (60). Pharmacological inhibition of DAT is known to consistently alter the autophagy machinery, whereas behavioral and neurotoxic effects can be overcome be autophagy blockade (61, 62). In the present study, hyperdopaminergic DAT-KO rats had detectably altered autophagy processes. However, it remains to be shown if the changes of individual proteins from a striatal comparative proteome are causally involved in the behavioral pattern of DAT-KO animals.

## Conclusion

Taken together, our behavioral findings demonstrate that the acquisition of new operant responses related to CS seems to be very sensitive to alterations of DA neurotransmission induced by the DAT depletion. The mechanisms underlying these findings are still elusive. However, we can speculate that hyperdopaminergic conditions result in the aberrant assignment of motivational salience to stimuli. Based on proteomic quantification results, we can propose that the downregulation of DRD1 could be responsible for the behavioral alterations here reported.

## Data Availability Statement

The datasets presented in this study can be found in online repositories. The names of the repository/repositories and accession number(s) can be found at: PRIDE, PXD028157.

## Ethics Statement

The animal study was reviewed and approved by the Local Animal Care and Use committee of First Pavlov State Saint Petersburg Medical University.

## Author Contributions

AS, JL, IS, and GL have written the manuscript. AS and IS carried out and analyzed the results of animal experiments. CM, JL, VK, LA-S, JM, and FS analyzed proteomic data. DL, IS, and GL initiated and designed the experiments. All authors contributed to the article and approved the submitted version.

## Conflict of Interest

The authors declare that the research was conducted in the absence of any commercial or financial relationships that could be construed as a potential conflict of interest.

## Publisher's Note

All claims expressed in this article are solely those of the authors and do not necessarily represent those of their affiliated organizations, or those of the publisher, the editors and the reviewers. Any product that may be evaluated in this article, or claim that may be made by its manufacturer, is not guaranteed or endorsed by the publisher.
